# Metabolic acidosis as a risk factor for bronchopulmonary dysplasia in preterm infants born between 23 + 0 and 31 + 6 weeks of gestation: a retrospective case-control study

**DOI:** 10.3389/fped.2025.1595348

**Published:** 2025-06-19

**Authors:** T. W. Shin, E. J. Lee, H. W. Choi, Y. M. Yoo

**Affiliations:** ^1^ Department of Pediatrics, Yonsei University Wonju College of Medicine, Wonju, Republic of Korea; ^2^ Department of Preventive Medicine, Yonsei University Wonju College of Medicine, Wonju, Republic of Korea

**Keywords:** metabolic acidosis, bronchopulmonary dysplasia, preterm infants, fluid balance, respiratory support

## Abstract

**Background:**

Metabolic acidosis is a common condition in preterm infants; however, its independent role in the development of bronchopulmonary dysplasia (BPD) remains unclear. In this study, we examined the association between metabolic acidosis and the occurrence of moderate/severe BPD or mortality in preterm infants born between 23 + 0 and 31 + 6 weeks of gestation.

**Methods:**

We retrospectively reviewed the medical records of 254 preterm infants (<32 weeks gestation) admitted between January 2017 and December 2024. The primary outcome was moderate/severe BPD or mortality before 36 weeks of postmenstrual age. Blood gas parameters, including pH, base excess, bicarbonate, and lactate, were analyzed daily during the first 14 days of life. Inverse probability of treatment weighting (IPTW) was applied to adjust for confounders such as gestational age, fluid balance, respiratory support, and patent ductus arteriosus (PDA) status.

**Results:**

After excluding infants with missing data (*n* = 88), 168 infants were included, of whom 55 developed moderate/severe BPD or died. Following IPTW adjustment, metabolic acidosis on day 6 of life (DOL 6) was significantly associated with moderate/severe BPD or mortality (OR: 1.369, 95% CI: 1.085–1.727). Differences in cumulative fluid intake were statistically significant during the first week (7.365% vs. 23.478%, *p* = 0.022) and became more pronounced in the second week after IPTW adjustment (8.206% vs. 22.888%, *p* = 0.024).

**Conclusion:**

Metabolic acidosis on DOL 6 was associated with an increased risk of moderate/severe BPD or mortality, suggesting its potential role as a modifiable risk factor. While excessive fluid intake has been linked to BPD, our findings highlight the complexity of fluid management in preterm infants. Further research is needed to determine whether correcting metabolic acidosis could improve respiratory outcomes.

## Graphical Abstract

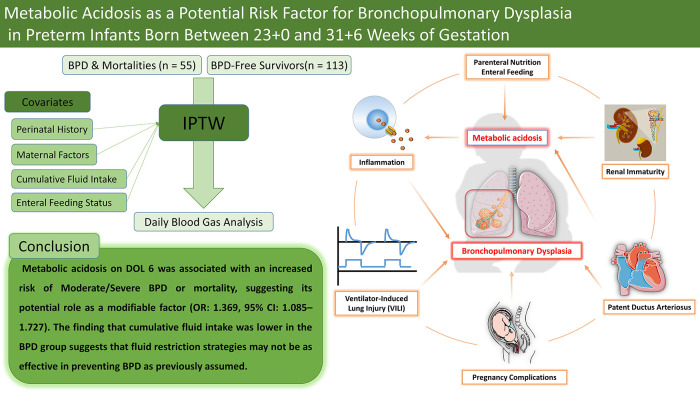

## 1 Introduction

Metabolic acidosis, characterized by decreased blood pH and bicarbonate levels due to an imbalance between acid production and elimination, occurs in approximately 18 per 1,000 infants born between 24 + 0 and 31 + 6 weeks of gestation, with its incidence increasing as gestational age decreases (1). It frequently develops within the first days of life due to multiple contributing factors, necessitating careful evaluation and management by neonatologists (2). In preterm infants, metabolic acidosis is primarily associated with immature renal function. A low glomerular filtration rate at birth, combined with reduced tubular function, leads to impaired bicarbonate reabsorption and decreased excretion of titratable acids and ammonium salts (3, 4).

Additionally, metabolic acidosis often occurs secondary to pathological conditions such as asphyxia, cardiovascular insufficiency, intraventricular hemorrhage (IVH), infection, and inborn errors of metabolism rather than as an isolated disorder (2, 5–7). It can also be exacerbated by early parenteral nutrition containing amino acids, lipids, and sodium chloride, as well as medications such as dopamine (8–10). Moreover, metabolic acidosis frequently coexists with respiratory acidosis, which results from impaired respiratory function or conditions such as respiratory distress syndrome (RDS) (11). This overlap complicates the determination of whether metabolic acidosis is a primary driver of disease or merely a marker of its severity. Consequently, its precise role in disease pathophysiology and the potential benefits of its correction remain uncertain (12, 13).

Bronchopulmonary dysplasia (BPD) is a common and significant complication in preterm infants, with its prevalence increasing at lower gestational ages (14). BPD develops through a multifactorial process involving various risk factors, including fetal growth restriction (15), antenatal and postnatal infections (16, 17), ventilator-induced lung injury (18), prolonged oxygen exposure (19), patent ductus arteriosus (17), and a genetic predisposition (20). Recent studies suggest that metabolic acidosis may contribute to BPD pathogenesis, providing new insights into its complex etiology. Observational studies have reported an association between early metabolic acidosis and an increased risk of BPD, while a randomized controlled trial (RCT) suggests that correcting metabolic acidosis may reduce its incidence (21, 22).

Although these studies have improved our understanding of metabolic acidosis in preterm infants, its diverse and multifactorial causes necessitate a more comprehensive evaluation of its impact on BPD. Building on existing evidence, this study aims to investigate the association between metabolic acidosis and the risk of moderate/severe BPD or mortality in preterm infants born before 32 weeks of gestation, while accounting for a broader range of contributing factors and potential confounders. Specifically, we seek to identify the critical period during which metabolic acidosis has the greatest impact on these outcomes.

## 2 Materials and methods

### 2.1 Study population

**Figure 1:**
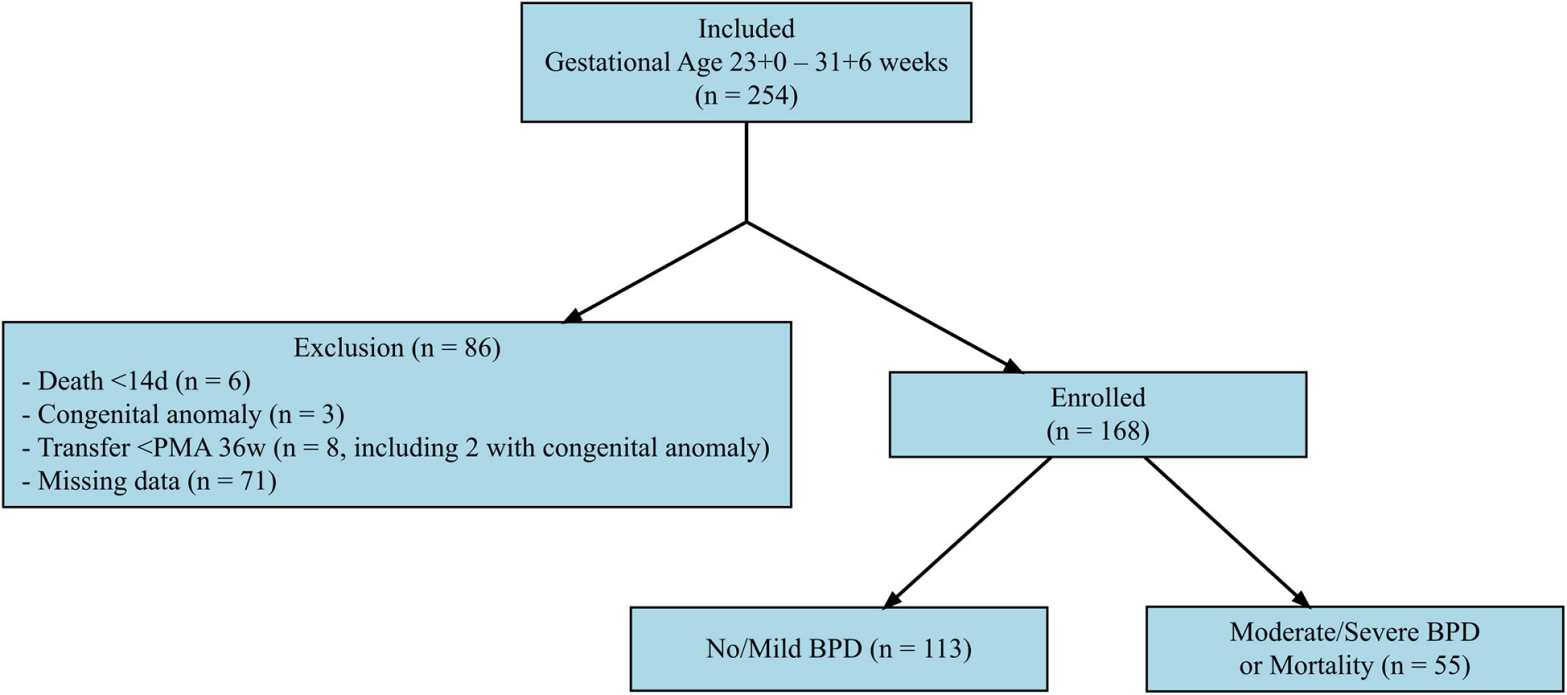
Selection process of the study population. This flowchart illustrates the selection process of the study population. A total of 254 preterm infants born between 23 + 0 and 31 + 6 weeks of gestation were initially assessed for eligibility. Of these, 86 infants were excluded due to death within the first 14 days of life (*n* = 6), congenital anomalies (*n* = 3), transfer to another hospital before reaching a postmenstrual age (PMA) of 36 weeks (*n* = 8, including 2 with congenital anomalies), or missing laboratory data (*n* = 71). The final study population consisted of 168 infants, who were categorized into two groups based on their primary outcome: no/mild bronchopulmonary dysplasia (BPD) (*n* = 113) and moderate-to-severe BPD or mortality (*n* = 55).

This retrospective case-control study was conducted in the neonatal intensive care unit of Wonju Severance Christian Hospital, a tertiary referral center in South Korea, between January 2017 and December 2024. The study included preterm infants born and admitted to our hospital with a gestational age between 23 + 0 and 31 + 6 weeks. Of the 254 infants screened, 86 were excluded due to early death within the first 14 days (*n* = 6), congenital anomalies (*n* = 3), transfer to another hospital before reaching a postmenstrual age of 36 weeks (*n* = 8, including 2 with congenital anomalies), or missing laboratory data (*n* = 71). Ultimately, 168 infants were included in the final analysis (Figure 1).

### 2.2 Data collection and measurements

Blood gas and electrolyte levels were measured daily using the i-STAT1 Analyzer (Abbott, MN: 300-G). The collected parameters included pH, standardized bicarbonate (mmol/L), base excess (mmol/L), lactate (mmol/L), sodium (mmol/L), chloride (mmol/L), and potassium (mmol/L). If multiple blood samples were obtained within a single day, the sample closest to a 24 hour interval from the previous test was selected for daily sampling. Medical records were reviewed to collect data on daily nutritional status and fluid intake, including enteral feeding volume (ml/kg/day) and total fluid intake (mL/kg/day), which encompassed both enteral and parenteral nutrition.

### 2.3 Definitions

#### 2.3.1 Outcome

The outcome of this study was moderate/severe bronchopulmonary dysplasia (BPD) or mortality before 36 weeks of postmenstrual age (PMA). BPD was defined according to the 2001 National Institutes of Health (NIH) consensus guidelines, which classify disease severity based on the need for respiratory support at 36 weeks PMA or at discharge, whichever occurred first, in preterm infants born before 32 weeks of gestation (23). Moderate BPD was defined as requiring supplemental oxygen for at least 28 days, with a continued oxygen requirement of <30% at 36 weeks PMA or discharge. Mortality was defined as death occurring before 36 weeks PMA or prior to discharge, assuming these cases were at least equivalent in severity to moderate/severe BPD for outcome analysis.

#### 2.3.2 Metabolic acidosis

Metabolic acidosis was defined as blood pH <7.35, with either base excess (BE) < −4 mmol/L or standardized bicarbonate (${\mathrm{HCO}}_{3}^{-}$ ) <18 mmol/L. Given the variability in definitions of metabolic acidosis among preterm infants across studies (9, 24, 25), we adopted a relatively broad to capture a wide range of acid-base disturbances. To complement this approach, we conducted a supplementary analysis using a stricter definition for severe metabolic acidosis to assess the impact of more profound metabolic acidosis. Metabolic acidosis status was assessed daily, and its presence was determined for each day of life based on these criteria.

#### 2.3.3 Covariates

The analysis included various neonatal and maternal variables as covariates. Neonatal variables included the 5-minute Apgar score, categorized as low if the score was <7, indicating neonatal depression and potential perinatal complications. Birth plurality was classified as singleton or non-singleton, while small for gestational age (SGA) was defined as a birth weight below the 10th percentile for gestational age based on the Fenton 2013 growth charts (26). Respiratory distress syndrome (RDS) was identified when at least one dose of surfactant was administered. Hemodynamically significant patent ductus arteriosus (hsPDA) was classified into two groups: infants without hsPDA or treatment and those who received medical or surgical intervention.

Nutritional factors included full enteral feeding, defined according to institutional criteria aligned with the ESPGHAN 2022 guidelines, achieved when an infant received >120 ml/kg/day of enteral nutrition for three consecutive days within the first 14 days of life (27). Cumulative fluid intake was calculated as the total fluid administered per kilogram of body weight per day and analyzed separately for the first and second weeks of life. The study population was divided into quartiles, and the highest quartile (Q4) for each period was assessed. The duration of invasive mechanical ventilation was measured as the total number of days an infant required endotracheal intubation within the first 14 days of life.

Maternal variables included assisted reproductive technology (ART), categorized based on the mode of conception as spontaneous or ART-assisted. Oligohydramnios was determined based on prenatal ultrasound findings. Preterm premature rupture of membranes (PPROM) was defined as membrane rupture lasting at least 18 h before delivery. Preeclampsia included cases diagnosed with chronic hypertension, gestational hypertension, preeclampsia, eclampsia, or HELLP syndrome. Gestational diabetes was defined as either overt diabetes mellitus or gestational diabetes. Antenatal steroid administration was considered present if the mother received at least one dose of prenatal corticosteroids.

### 2.4 Statistical analysis

Initial comparisons were conducted using an unweighted model to evaluate differences based on moderate/severe BPD or mortality. Gestational age, birth weight, duration of invasive mechanical ventilation (days) in the first 14 days, and maternal age were compared using *t*-tests, while sex, 5 min Apgar score (<7), single/multiple delivery, small for gestational age, RDS, hsPDA, ART, oligohydramnios, PPROM, preeclampsia, gestational diabetes mellitus (GDM), antenatal steroid administration, mode of delivery, achievement of full enteral feeding within 14 days, and cumulative fluid intake were compared using chi-square tests.

To adjust for covariates associated with the outcome, inverse probability of treatment weighting (IPTW) was applied. A binary logistic regression model was initially constructed with moderate/severe BPD or mortality as the dependent variable, while all covariates were included as independent variables. A stepwise selection method was used to identify the most explanatory model, which was then used to estimate the probability of moderate/severe BPD or mortality for each subject.

Based on these estimated probabilities, IPTW values were calculated. To minimize weight distortion, stabilized weights were derived to preserve the overall proportion of subjects with moderate/severe BPD or mortality. Weighted comparisons, including mean and proportion analyses, were subsequently performed on the stabilized sample.

To compare overall laboratory data related to the outcome, blood gas and electrolyte levels were analyzed over a 14-day period and compared between the moderate/severe BPD or mortality group and the no/mild BPD group. Comparisons were conducted using both unweighted and weighted means with standard deviations (mean ± SD). Additionally, daily trends in pH, base excess, bicarbonate, and lactate levels were visualized in a figure to illustrate their temporal changes in relation to the outcome.

To evaluate the impact of daily metabolic acidosis, weighted and unweighted logistic regression analyses were performed to assess the association between the presence of metabolic acidosis during days 1–14 of life and moderate/severe BPD or mortality. All analyses were also conducted using unweighted models for comparison.

As a sensitivity analysis, the primary analysis was repeated after excluding subjects who experienced mortality to assess the robustness of the findings. Additionally, the analysis was stratified into two periods (first week vs. second week of life) to examine period-specific effects.

For the subgroup analysis, the population was further stratified by gestational age, dividing subjects into those born before 28 weeks and those born at or after 28 weeks, to explore potential differences in outcomes between these groups.

Statistical analyses were performed using R version 4.3.3 (R Foundation), and a *p*-value < 0.05 was considered statistically significant. All statistical scripts used for this analysis are available at the following GitHub repository: https://github.com/hyoone1/MABPD.

### 2.5 Ethics statement

This study adhered to the principles of the Declaration of Helsinki and received approval from the Institutional Review Board of Wonju Severance Christian Hospital (CR324093).

## 3 Results

### 3.1 Baseline characteristics

The baseline characteristics of the study population before and after inverse probability of treatment weighting (IPTW) are presented in Table 1. The unweighted cohort included 55 infants with moderate/severe BPD or mortality and 113 infants with no/mild BPD.

**Table 1:** Baseline characteristics of study population before and after IPTW*.

Variables	Unweighted (mean ± SD or *N* (%))			Weighted (mean ± SD, or *N* (%))		
	Moderate/severe BPD or mortality (*N* = 55)	No/mild BPD (*N* = 113)	*p*-value	Moderate/severe BPD or mortality (*N* = 48.992)	No/mild BPD (*N* = 105.207)	*p*-value
Neonatal characteristics						
Gestational age (weeks)	25.9 ± 2.0	28.7 ± 1.7	**<0**.**001**	27.636 ± 0.705	28.089 ± 0.226	0.324
Birth weight (grams)	832.5 ± 291.2	1,255.0 ± 299.8	**<0**.**001**	1,097.285 ± 124.293	1,168.989 ± 33.279	0.386
Sex			0.568			0.441
Male	33 (60.0%)	61 (54.0%)		44.332%	56.017%	
Apgar score at 5 min <7	20 (36.4%)	27 (23.9%)	0.132	23.010%	30.444%	0.472
Twin birth	16 (34.8%)	35 (31.0%)	0.958	25.160%	28.620%	0.754
Small for gestational age	6 (10.9%)	3 (2.7%)	0.062	5.172%	3.546%	0.618
Respiratory distress syndrome of Newborn	51 (92.7%)	89 (78.8%)	**0**.**040**	91.620%	80.667%	0.154
Hemodynamically significant PDA (hsPDA)			**<0**.**001**			0.687
No hsPDA or No Treatment	21 (38.2%)	92 (81.4%)		64.043%	69.169%	
Treatment (medication or surgical ligation)	34 (61.8%)	21 (18.6%)		35.957%	30.831%	
Invasive Mechanical ventilation (days) in the first 14 days	10.6 ± 5.6	3.5 ± 4.7	**<0**.**001**	5.651 ± 1.769	5.199 ± 0.711	
Maternal characteristics						
Maternal age (years)	32.7 ± 4.9	32.9 ± 4.9	0.802	33.053 ± 0.597	32.953 ± 0.558	0.866
Assisted reproductive technology	14 (25.5%)	30 (26.5%)	1.000	15.090%	26.474%	0.176
Oligohydramnios	6 (10.9%)	15 (13.3%)	0.852	8.332%	11.932%	0.558
Preterm premature rupture of membranes	16 (29.1%)	44 (38.9%)	0.281	26.078%	36.544%	0.387
Preeclampsia	12 (21.8%)	15 (13.3%)	0.234	16.703%	15.107%	0.840
Gestational diabetes	5 (9.1%)	9 (8.0%)	1.000	6.775%	7.171%	0.932
Antenatal steroid (≥1 dose)	49 (89.1%)	93 (82.3%)	0.360	89.922%	84.016%	0.421
Cesarean section	48 (87.3%)	92 (81.4%)	0.462	66.109%	83.363%	0.287
Nutrition & fluid management						
Full enteral feeding (>120 ml/kg/day for 3 consecutive days within 14 days)	8 (14.5%)	60 (53.1%)	**<0**.**001**	48.022%	42.802%	0.754
Cumulative fluid intake (mL/kg/day), Q4						
During 1st week	6 (10.9%)	32 (28.3%)	**0**.**020**	7.365%	23.478%	**0**.**022**
During 2nd week	8 (14.5%)	30 (26.5%)	0.121	8.206%	22.888%	**0**.**024**

* IPTW, inverse probability of treatment weighting.

Bold values indicate *p* < 0.05.

Before IPTW, infants in the moderate/severe BPD or mortality group had significantly lower gestational age (25.9 ± 2.0 vs. 28.7 ± 1.7 weeks, *p* < 0.001) and birth weight (832.5 ± 291.2 vs. 1,255.0 ± 299.8 grams, *p* < 0.001) compared to those in the no/mild BPD group. RDS was more prevalent in the moderate/severe BPD or mortality group (92.7% vs. 78.8%, *p* = 0.040), and the proportion of infants with hsPDA requiring treatment was significantly higher (61.8% vs. 18.6%, *p* < 0.001). Additionally, these infants had a longer duration of mechanical ventilation in the first 14 days of life (10.6 ± 5.6 vs. 3.5 ± 4.7 days, *p* < 0.001).

After IPTW, the baseline characteristics between the two groups were well-balanced, with no statistically significant differences in gestational age (27.636 ± 0.705 vs. 28.089 ± 0.226 weeks, *p* = 0.324), birth weight (1,097.285 ± 124.293 vs. 1,168.989 ± 33.279 grams, *p* = 0.386), RDS prevalence (91.620% vs. 80.667%, *p* = 0.154), and hsPDA requiring treatment (35.957% vs. 30.831%, *p* = 0.687). However, differences in cumulative fluid intake during the first week persisted, with a higher proportion of infants in the moderate/severe BPD or mortality group belonging to the highest quartile (Q4) of fluid intake during the first week (7.365% vs. 23.478%, *p* = 0.022) and the second week (8.206% vs. 22.888%, *p* = 0.024).

### 3.2 Blood gas and electrolyte levels

Table 2 summarizes blood gas and electrolyte levels before and after IPTW. Before IPTW, infants in the moderate/severe BPD or mortality group exhibited significantly lower pH (7.281 ± 0.040 vs. 7.322 ± 0.039, *p* *<* 0.001) and bicarbonate levels (20.208 ± 2.420 vs. 21.589 ± 2.442 mmol/L, *p* *=* 0.001), along with higher chloride levels (114.421 ± 4.185 vs. 112.243 ± 3.916 mmol/L, *p* *=* 0.001), indicating a greater degree of metabolic acidosis. However, after IPTW, these differences were no longer statistically significant: pH (7.313 ± 0.016 vs. 7.314 ± 0.005, *p* *=* 0.970), bicarbonate levels (21.273 ± 0.526 vs. 21.282 ± 0.291 mmol/L, *p* *=* 0.988), and chloride levels (112.427 ± 0.744 vs. 112.559 ± 0.429 mmol/L, *p* *=* 0.879).

**Table 2:** Blood Gas and electrolyte levels in moderate/severe BPD or mortality vs. No/Mild BPD Groups.

Variables	Unweighted (mean ± SD or *N* (%))			Weighted (mean ± SD, or *N* (%))		
	Moderate/severe BPD or mortality *N* = 55)	No/mild BPD (*N* = 113)	*p*-value	Moderate/severe BPD or mortality (*N* = 48.992)	No/mild BPD (*N* = 105.207)	*p*-value
Blood gas parameters						
pH	7.281 ± 0.040	7.322 ± 0.039	**<0**.**001**	7.313 ± 0.016	7.314 ± 0.005	0.970
Base Excess, mmol/L	−6.762 ± 2.953	−4.608 ± 2.929	**<0**.**001**	−5.286 ± 0.683	−5.044 ± 0.356	0.755
Bicarbonate(Standardized), mmol/L	20.208 ± 2.420	21.589 ± 2.442	**0**.**001**	21.273 ± 0.526	21.282 ± 0.291	0.988
Lactate, mmol/L	3.722 ± 15.439	1.447 ± 0.391	0.280	2.417 ± 0.911	1.442 ± 0.042	0.287
Electrolytes, mmol/L						
Sodium	136.016 ± 3.020	135.838 ± 2.595	0.693	135.535 ± 0.297	135.837 ± 0.321	0.490
Chloride	114.421 ± 4.185	112.243 ± 3.916	**0**.**001**	112.427 ± 0.744	112.559 ± 0.429	0.879
Potassium	4.824 ± 0.422	4.835 ± 0.337	0.868	4.809 ± 0.056	4.788 ± 0.045	0.771
Anion Gap	6.538 ± 4.281	7.016 ± 3.781	0.463	6.684 ± 0.473	6.882 ± 0.726	0.743

Bold values indicate *p* < 0.05.

Additionally, the anion gap remained comparable between the two groups both before (6.538 ± 4.281 vs. 7.016 ± 3.781, *p* *=* 0.463) and after IPTW adjustment (6.684 ± 0.473 vs. 6.882 ± 0.726, *p* *=* 0.743).

**Figure 2:**
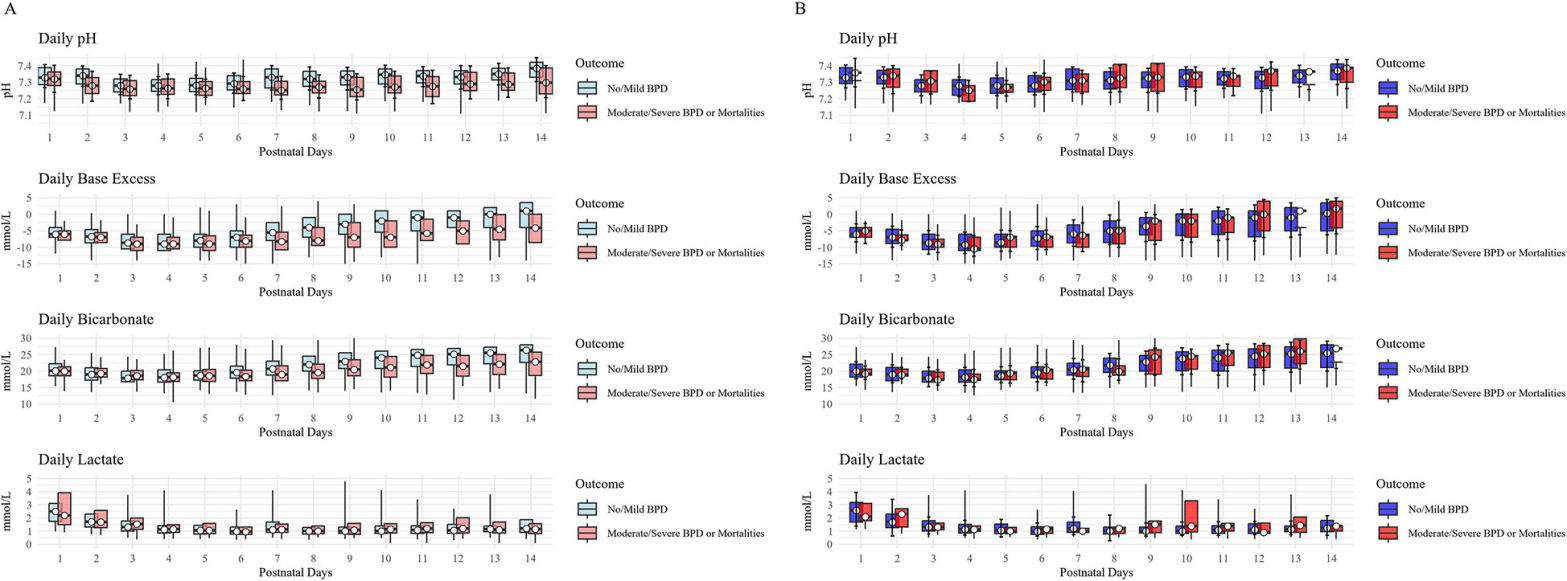
**(A,B)** trends in blood Gas parameters before and after IPTW. This figure presents the daily trends of blood gas parameters, including pH, base excess (BE), bicarbonate (${\mathrm{HCO}}_{3}^{-}$), and lactate levels, over the first 14 postnatal days in preterm infants. The study population was categorized into two groups based on the primary outcome: no/mild bronchopulmonary dysplasia (BPD) (light blue) and moderate/severe BPD or mortality (light coral). The box plots represent the interquartile range (IQR; 25th–75th percentile), with whiskers extending to the minimum and maximum observed values. The white circles indicate the median values, and error bars represent the mean ± standard deviation (SD). **(A)** displays trends before IPTW, while **(B)** presents trends after IPTW adjustment. These trends illustrate differences in acid-base balance and metabolic status between the two groups before and after inverse probability of treatment weighting (IPTW) was applied.

The daily trends of blood gas and electrolyte levels over the first 14 days are illustrated in Figure 2. Before IPTW, infants in the moderate/severe BPD or mortality group consistently exhibited lower pH, lower bicarbonate levels, and more negative base excess compared to those with no/mild BPD. However, after IPTW adjustment, these differences were largely attenuated.

### 3.3 Association between metabolic acidosis and outcomes

The association between metabolic acidosis and the primary outcome was evaluated using generalized linear models (GLM) before and after IPTW (Table 3). Before IPTW, acidosis on DOL 2 was significantly associated with an increased risk of Moderate/Severe BPD or mortality (OR: 1.167; 95% CI: 1.006–1.353). However, this association lost significance after IPTW adjustment (OR: 0.994; 95% CI: 0.843–1.172).

**Table 3:** Metabolic acidosis and outcome: unweighted vs. IPTW Model*.

Acidosis occurrence	With acidosis *n* (%)	Without acidosis *n* (%)	Unweighted model (GLM) OR (95% CI)	IPTW model OR (95% CI)
DOL** 1	72 (42.9%)	96 (57.1%)	1.050 (0.912–1.210)	1.011 (0.858–1.191)
DOL 2	93 (55.4%)	75 (44.6%)	**1.167** (**1.006–1.353)**	0.994 (0.843–1.172)
DOL 3	133 (79.2%)	35 (20.8%)	0.931 (0.778–1.115)	0.786 (0.593–1.041)
DOL 4	140 (83.3%)	28 (16.7%)	0.887 (0.725–1.085)	0.981 (0.765–1.259)
DOL 5	134 (79.8%)	34 (20.2%)	0.948 (0.765–1.175)	1.030 (0.802–1.322)
DOL 6	120 (71.4%)	48 (28.6%)	1.161 (0.932–1.446)	**1.369** (**1.085–1.727)**
DOL 7	102 (60.7%)	66 (39.3%)	1.064 (0.866–1.306)	0.865 (0.68–1.101)
DOL 8	86 (51.2%)	82 (48.8%)	0.889 (0.724–1.090)	0.860 (0.707–1.046)
DOL 9	75 (44.6%)	93 (55.4%)	1.249 (0.997–1.565)	1.210 (0.976–1.500)
DOL 10	68 (40.5%)	100 (59.5%)	1.107 (0.858–1.428)	1.271 (0.939–1.720)
DOL 11	60 (35.7%)	108 (64.3%)	0.895 (0.690–1.160)	0.802 (0.604–1.065)
DOL 12	54 (32.1%)	114 (67.9%)	1.063 (0.825–1.370)	1.052 (0.817–1.353)
DOL 13	49 (29.2%)	119 (70.8%)	1.065 (0.822–1.379)	1.001 (0.774–1.295)
DOL 14	46 (27.4%)	122 (72.6%)	1.075 (0.834–1.387)	0.963 (0.729–1.271)

Bold values indicate *p* < 0.05.

* IPTW, inverse probability of treatment weighting; **DOL, day(s) of life.

In contrast, acidosis on DOL 6, which was not significant before IPTW (OR: 1.161; 95% CI: 0.932–1.446), became significantly associated after IPTW adjustment (OR: 1.369; 95% CI: 1.085–1.727).

No other significant associations were observed on other days, with odds ratios ranging from 0.786 to 1.271, none of which reached statistical significance.

### 3.4 Sensitivity analysis

#### 3.4.1 Excluding mortality (Supplementary Table S1)

To determine whether the observed association between metabolic acidosis and BPD was independent of mortality, a sensitivity analysis was conducted excluding mortality cases. Acidosis on DOL 6 remained significantly associated with moderate/severe BPD even after IPTW adjustment (OR: 1.251; 95% CI: 1.010–1.550). No significant associations were observed on other days.

#### 3.4.2 Weekly metabolic acidosis (Supplementary Table S2)

The impact of metabolic acidosis during the first and second weeks of life was examined. Before IPTW, metabolic acidosis during the second week was significantly associated with the primary outcome (OR: 1.289; 95% CI: 1.114–1.491), but this association lost significance after IPTW adjustment (OR: 0.924; 95% CI: 0.671–1.273). No significant associations were found for first-week metabolic acidosis in either model.

#### 3.4.3 Severe metabolic acidosis (Supplementary Table S3)

To evaluate whether the severity of metabolic acidosis affected the outcome, we conducted a sensitivity analysis using a stricter definition of severe metabolic acidosis (pH < 7.20 and either base excess < –10 mmol/L or standardized bicarbonate < 12 mmol/L), as used by Notz et al. (21). In the IPTW model, severe acidosis on DOL 1, 4, 7, and 11 was significantly associated with an increased risk of Moderate/Severe BPD (DOL 1: OR 2.508, 95% CI: 1.947–3.231; DOL 4: OR 1.738, 95% CI: 1.262–2.394; DOL 7: OR 1.977, 95% CI: 1.665–2.347).

### 3.5 Subgroup analysis

In the subgroup analysis, 128 infants were born at <28 weeks of gestation and 102 were born at 28–31 weeks. Among infants born before 28 weeks, 40 (60.6%) developed moderate/severe BPD or mortality, while 26 (39.4%) did not. In contrast, among those born at 28–31 weeks, only 15 (14.7%) experienced moderate/severe BPD or mortality, whereas 87 (85.3%) did not.

#### 3.5.1 Infants born before 28 Weeks' gestation (Supplementary Table S4)

A subgroup analysis was conducted for infants born before 28 weeks of gestation. After IPTW, none of the time points showed a statistically significant association between metabolic acidosis and moderate/severe BPD or mortality. The odds ratios fluctuated across different days, with the highest observed on DOL2 (OR: 1.293; 95% CI: 0.976–1.713), though it did not reach statistical significance.

#### 3.5.2 Infants born at 28–31 Weeks' gestation (Supplementary Table S5)

For infants born between 28 and 31 weeks of gestation, after IPTW, metabolic acidosis on Days 6 and 9 was significantly associated with moderate/severe BPD or mortality (DOL 6: OR: 1.226; 95% CI: 1.015–1.482; DOL 9: OR: 1.263; 95% CI: 1.024–1.559). Acidosis on other days did not demonstrate significant associations with the outcome in this subgroup.

## 4 Discussion

In this study of preterm infants born between 23 + 0 and 31 + 6 weeks of gestation, we found that metabolic acidosis on DOL 6 was associated with an increased risk of moderate/severe BPD or mortality, even after adjusting for confounding variables using inverse probability of treatment weighting (IPTW). Additionally, infants in the moderate/severe BPD or mortality group had lower cumulative fluid intake during both the first and second weeks of life. These findings provide further insight into the relationship between metabolic acidosis and BPD in preterm infants.

First, our results align with previous research demonstrating a link between metabolic acidosis and BPD in preterm infants. Notz et al. observed that metabolic acidosis during the first week of life was significantly associated with an increased risk of BPD or death in infants born before 28 weeks' gestation, suggesting that metabolic acidosis could serve as an early marker for moderate/severe BPD or mortality (21). Similarly, in our cohort (<32 weeks), metabolic acidosis on DOL 6 remained significantly associated with BPD or mortality after adjusting for confounders, reinforcing this potential link. However, it is noteworthy that among the 14 postnatal days evaluated, only DOL 6 showed a statistically significant association. This isolated finding should be interpreted with caution, as it may reflect a chance association or an indirect signal influenced by other underlying conditions. The absence of consistent associations across other time points suggests that metabolic acidosis may not function as a standalone predictor of BPD, but rather as one component within a broader, multifactorial risk framework.

Unlike Notz et al., our study did not find a significant association between metabolic acidosis and BPD in infants born before 28 weeks' gestation, suggesting that other factors, such as oxidative stress, prolonged mechanical ventilation, and hemodynamically significant PDA, may have a greater influence on BPD risk in this group. Instead, the association was more pronounced in infants born at 28–31 weeks, indicating that metabolic acidosis may be more relevant in relatively more mature preterm infants, reflecting potential gestational age-dependent differences in BPD pathophysiology. While Notz et al. used a severe metabolic acidosis definition (pH < 7.2 with BE < −10 mmol/L or SBC < 12 mmol/L), our study employed a more lenient threshold (pH < 7.35, BE < −4 mmol/L, or SBC < 18 mmol/L) yet still observed a similar association. To further explore this difference in definition, we conducted a sensitivity analysis using the stricter criteria proposed by Notz et al. Interestingly, in the IPTW model, severe metabolic acidosis occurring predominantly during the first postnatal week was significantly associated with Moderate/Severe BPD, a finding that aligns with the results of Notz et al., highlighting that the severity of acid-base imbalance—rather than its mere presence—may be a more critical determinant of adverse respiratory outcomes in preterm infants. By adjusting for a broader range of neonatal, maternal, and gestational factors using IPTW, our analysis provides a refined assessment of the independent effect of metabolic acidosis on BPD risk. The findings remained robust even after excluding mortality cases. Given the complex interplay between metabolic acidosis and other clinical factors, caution is needed in interpretation, and further research is warranted to clarify potential causal relationships.

Second, our study found that cumulative fluid intake was lower in the moderate/severe BPD or mortality group, with fewer infants in the highest quartile (Q4). This is despite previous studies reporting that higher fluid intake is associated with an increased risk of BPD in preterm infants, as excessive fluid administration is well known to contribute to pulmonary edema and impaired lung function (28–30). administration is well known to contribute to pulmonary edema and impaired lung function (26–28). This discrepancy may be explained by differences in the intensity of fluid management strategies. Although a fluid restriction protocol is applied to all infants under 32 weeks' gestation in our institution, more stringent fluid limitation is often practiced in those born before 28 weeks due to their higher vulnerability to complications such as hsPDA and prolonged respiratory support. Therefore, the lower fluid intake observed in the Moderate/Severe BPD or mortality group may reflect this risk-adapted clinical practice. Despite fluid restriction, BPD still developed, suggesting that factors beyond fluid management—such as low gestational age, prolonged mechanical ventilation, and hsPDA—play a more significant role in BPD pathogenesis, a notion further supported by the persistence of significant fluid intake differences between groups even after IPTW adjustment in both the first and second weeks. Rather than fluid intake acting as a direct causal factor, it may primarily reflect clinical management strategies tailored to high-risk infants. In support of this interpretation, our supplementary analysis showed that daily fluid intake was significantly higher on days with metabolic acidosis, and this may reflect therapeutic interventions such as fluid boluses or bicarbonate administration aimed at correcting acidosis (Supplementary Table S6 and Supplementary Figure S1).

While previous studies have suggested that excessive fluid intake increases BPD risk, our findings raise the possibility that strict fluid restriction alone may not necessarily be protective against BPD. This underscores the need for a more nuanced approach in evaluating the role of fluid management in preterm lung disease, considering a broader range of clinical factors.

Third, this study also examined the impact of enteral nutrition volume, which may help mitigate metabolic acidosis and influence BPD risk. Since parenteral nutrition contributes to metabolic acidosis in preterm infants (31, 32), higher enteral intake may reduce acidosis by decreasing reliance on parenteral nutrition. In line with this, some studies have suggested a relationship between enteral feeding and BPD (33, 34). In our study, before IPTW, infants in the moderate/severe BPD or mortality group had significantly lower rates of achieving full enteral feeding within 14 days compared to those with no/mild BPD. However, after IPTW adjustment, this difference was no longer significant. This highlights the challenge of assessing the impact of enteral feeding on acidosis and BPD risk, as early postnatal instability and comorbidities significantly influence the initiation and advancement of enteral feeding. Given these complexities, further research is needed to better delineate the independent role of enteral feeding in metabolic and respiratory outcomes.

Regarding laboratory findings (Table 2), before IPTW, the moderate/severe BPD or mortality group exhibited significantly lower pH, base excess, and bicarbonate levels, along with higher chloride levels, indicating a greater degree of metabolic acidosis. These findings are consistent with previous research (21). However, after IPTW adjustment, all differences lost statistical significance. This aligns with our overall study findings, suggesting that the impact of metabolic acidosis on BPD risk is largely mediated by other clinical variables rather than acting as a primary driver of disease.

### 4.1 Strengths

This study accounts for key factors such as fluid intake, enteral feeding, and invasive ventilation duration, providing deeper insight into the interplay between metabolic acidosis and neonatal outcomes. Building on existing research, we employed inverse probability of treatment weighting (IPTW) to rigorously adjust for confounders, allowing for a more precise assessment of metabolic acidosis as an independent risk factor for BPD. Furthermore, the robustness of our findings was reinforced through sensitivity and subgroup analyses, ensuring the validity of our conclusions across different clinical contexts. Additionally, by adopting a more inclusive definition of metabolic acidosis, our study offers a broader perspective that may enhance its explanatory power compared to previous research.

### 4.2 Limitations

This study's retrospective, single-center design and relatively small sample size may have introduced selection bias. Additionally, blood gas analysis was performed using capillary sampling, which may differ from arterial blood gas analysis. However, the use of a consistent sampling method and analyzer throughout the study period likely mitigated this limitation. Furthermore, a higher-than-expected number of patients (*n* = 71) had missing laboratory data and were excluded. To assess potential bias, we compared data before and after excluding missing cases. Although the proportion of moderate-to-severe BPD was slightly lower in the missing data group compared to the non-missing group (24.9% vs. 32.7%), this difference was not statistically significant (*χ*^2^ = 2.62, *p* = 0.106), suggesting that the missing data were non-differential. In addition, we used the 2001 NIH definition of BPD, which is now considered outdated. Although still widely used, the 2019 Jensen definition (35) may offer better clinical relevance. Future studies should consider adopting the updated criteria.

## 5 Conclusion

Metabolic acidosis on DOL 6 was associated with an increased risk of moderate/severe BPD or mortality, even after adjusting for clinical factors such as gestational age, fluid balance, enteral feeding, respiratory support, and hemodynamically significant PDA. However, this association was not consistently observed across other postnatal days, suggesting that metabolic acidosis may reflect an interaction with other clinical risk factors rather than serving as a primary causal agent. Rather than being a mere physiological phenomenon, metabolic acidosis may represent a modifiable factor in neonatal outcomes. While excessive fluid intake has been linked to BPD risk, our findings suggest that the impact of fluid restriction strategies on BPD may be more complex than previously understood. Further research is needed to elucidate the underlying mechanisms and causality of metabolic acidosis in BPD development, as well as to evaluate the potential benefits of metabolic acidosis correction.

## Data Availability

The data analyzed in this study is subject to the following licenses/restrictions: The datasets generated and analyzed during this study are not publicly available due to participant privacy concerns. Requests for data access can be directed to YM Yoo, the corresponding author. Requests to access these datasets should be directed to Yeong Myong Yoo, ym3023822@yonsei.ac.kr.
