# Supplementary material for: Metabolic acidosis as a risk factor for bronchopulmonary dysplasia in preterm infants born between 23 + 0 and 31 + 6 weeks of gestation: a retrospective case-control study
Source: Front Pediatr. 2025 Jun 19;13:1595348. doi: 10.3389/fped.2025.1595348 (PMC12222276; doi:10.3389/fped.2025.1595348)
Supplement: Supplementary file 1 [file Supplementaryfile1.docx]

Supplementary Materials

**Supplementary Table 1. Sensitivity Analysis : Metabolic Acidosis and Outcome : Unweighted vs. IPTW* Model (Excluding Mortalities)**

| **Acidosis Occurrence** | **With Acidosis**  **n (%)** | **Without Acidosis**  **n (%)** | **Unweighted Model(GLM**)**  **OR (95% CI)** | **IPTW Model**  **OR (95% CI)** |
| --- | --- | --- | --- | --- |
| DOL** 1 | 64 (40.3%) | 95 (59.7%) | 1.001 (0.867-1.157) | 0.993 (0.850-1.162) |
| DOL 2 | 86 (54.1%) | 73 (45.9%) | **1.179 (1.013-1.372)** | 1.020 (0.871-1.196) |
| DOL 3 | 125 (78.6%) | 34 (21.4%) | 0.932 (0.776-1.118) | 0.838 (0.640-1.098) |
| DOL 4 | 133 (83.6%) | 26 (16.4%) | 0.898 (0.730-1.106) | 0.973 (0.766-1.236) |
| DOL 5 | 126 (79.2%) | 33 (20.8%) | 0.942 (0.756-1.174) | 1.019 (0.804-1.291) |
| DOL 6 | 112 (70.4%) | 47 (29.6%) | 1.170 (0.931-1.470) | **1.251 (1.010-1.550)** |
| DOL 7 | 93 (58.5%) | 66 (41.5%) | 1.032 (0.840-1.267) | 0.914 (0.733-1.141) |
| DOL 8 | 78 (49.1%) | 81 (50.9%) | 0.895 (0.724-1.106) | 0.897 (0.748-1.077) |
| DOL 9 | 67 (42.1%) | 92 (57.9%) | 1.234 (0.975-1.561) | 1.187 (0.964-1.463) |
| DOL 10 | 60 (37.7%) | 99 (62.3%) | 1.063 (0.817-1.382) | 1.191 (0.875-1.622) |
| DOL 11 | 54 (34%) | 105 (66%) | 0.902 (0.691-1.178) | 0.816 (0.618-1.077) |
| DOL 12 | 49 (30.8%) | 110 (69.2%) | 1.069 (0.813-1.405) | 1.039 (0.819-1.320) |
| DOL 13 | 44 (27.7%) | 115 (72.3%) | 1.097 (0.834-1.442) | 1.013 (0.772-1.330) |
| DOL 14 | 40 (25.2%) | 119 (74.8%) | 1.031 (0.794-1.339) | 0.947 (0.707-1.268) |

Sensitivity analysis of the association between metabolic acidosis and adverse outcomes by postnatal day of life, excluding mortalities. Both unweighted generalized linear models (GLM) and inverse probability of treatment weighting models were used to estimate the odds ratios (ORs) and 95% confidence intervals (CIs) for each day. The analysis compares outcomes between infants with and without metabolic acidosis on each respective day. IPTW models were adjusted for baseline covariates to minimize confounding. Significant association was observed only on DOL 6 in the IPTW model.
*IPTW, Inverse Probability of Treatment Weighting; **GLM, generalized linear model; DOL, Day(s) of life

**Supplementary Table 2. Sensitivity Analysis : Impact of First and Second Week Metabolic Acidosis on Outcome: Unweighted vs. IPTW Model**

|  | **With Acidosis**  **n (%)** | **Without Acidosis**  **n (%)** | **Unweighted Model(GLM)**  **OR (95% CI)** | **IPTW Model**  **OR (95% CI)** |
| --- | --- | --- | --- | --- |
| Acidosis_Week 1 | 163 (97.0%) | 5 (0.03%) | 0.970 (0.639-1.472) | 1.129 (0.678-1.882) |
| Acidosis_Week 2 | 104 (61.9%) | 64 (38.1%) | **1.289 (1.114-1.491)** | 0.924 (0.671-1.273) |

Sensitivity analysis examining the association between metabolic acidosis during the first and second postnatal weeks and adverse outcomes. Week 1 includes any occurrence of metabolic acidosis from DOL 1 to 7, and Week 2 includes occurrences from DOL 8 to 14. While unweighted analysis suggested a significant association during Week 2, the IPTW-adjusted model did not support this finding.

**Supplementary Table 3. Severe Metabolic Acidosis and Outcome : Unweighted vs. IPTW* Model (Excluding Mortalities)**

| **Acidosis Occurrence** | **With Acidosis**  **n (%)** | **Without Acidosis**  **n (%)** | **Unweighted Model(GLM)**  **OR (95% CI)** | **IPTW Model**  **OR (95% CI)** |
| --- | --- | --- | --- | --- |
| DOL** 1 | 1 (0.6%) | 167 (99.4%) | 2.282 (0.719-7.241) | **2.508 (1.947-3.231)** |
| DOL 2 | 4 (2.4%) | 164 (97.6%) | 1.028 (0.536-1.970) | 1.104 (0.726-1.678) |
| DOL 3 | 7 (4.2%) | 161 (95.8%) | 1.059 (0.744-1.509) | 0.907 (0.717-1.148) |
| DOL 4 | 11 (6.5%) | 157 (93.5%) | 1.211 (0.902-1.626) | **1.738 (1.262-2.394)** |
| DOL 5 | 9 (5.4%) | 159 (94.6%) | 1.045 (0.740-1.476) | 0.930 (0.644-1.342) |
| DOL 6 | 6 (3.6%) | 162 (96.4%) | 0.899 (0.558-1.447) | 0.736 (0.471-1.150) |
| DOL 7 | 6 (3.6%) | 162 (96.4%) | **1.804 (1.200-2.714)** | **1.977 (1.665-2.347)** |
| DOL 8 | 7 (4.2%) | 161 (95.8%) | 1.042 (0.696-1.560) | 0.928 (0.706-1.221) |
| DOL 9 | 6 (3.6%) | 162 (96.4%) | 0.823 (0.538-1.260) | 0.769 (0.542-1.090) |
| DOL 10 | 6 (3.6%) | 162 (96.4%) | 1.312 (0.816-2.109) | 1.375 (0.983-1.924) |
| DOL 11 | 7 (4.2%) | 161 (95.8%) | 1.545 (0.970-2.461) | **1.637 (1.204-2.226)** |
| DOL 12 | 6 (3.6%) | 162 (96.4%) | 0.890 (0.533-1.487) | 0.773 (0.437-1.369) |
| DOL 13 | 4 (2.4%) | 164 (97.6%) | 1.204 (0.690-2.102) | 1.380 (0.883-2.157) |
| DOL 14 | 2 (1.2%) | 166 (98.8%) | 1.055 (0.472-2.359) | 1.019 (0.650-1.597) |

Sensitivity analysis evaluating the association between severe metabolic acidosis and adverse outcomes by day of life (DOL, excluding mortalities. Severe metabolic acidosis was defined as arterial pH < 7.20 accompanied by either base excess ≤ –10 mmol/L or standardized bicarbonate < 12 mmol/L, based on criteria by Notz et al. Notably, IPTW-adjusted models showed significant associations on DOL 1, DOL 4, DOL 7, and DOL 11.
*IPTW, Inverse Probability of Treatment Weighting; **DOL, Day(s) of life

**Supplementary Table 4. Subgroup Analysis: Association Between Metabolic Acidosis and Outcome in ELGAN (<28 Weeks' GA)**

| **Acidosis** **Occurrence** | **With Acidosis**  **n (%)** | **Without Acidosis**  **n (%)** | **Unweighted Model(GLM)**  **OR (95% CI)** | **IPTW* Model**  **OR (95% CI)** |
| --- | --- | --- | --- | --- |
| DOL** 1 | 32 (48.5%) | 34 (51.5%) | 1.201 (0.927-1.555) | 1.138 (0.887-1.460) |
| DOL 2 | 51 (77.3%) | 15 (22.7%) | 1.220 (0.893-1.665) | 1.293 (0.976-1.713) |
| DOL 3 | 57 (86.4%) | 9 (13.6%) | 1.025 (0.691-1.520) | 1.087 (0.773-1.528) |
| DOL 4 | 58 (87.9%) | 8 (12.1%) | 0.824 (0.543-1.252) | 0.806 (0.591-1.101) |
| DOL 5 | 56 (84.8%) | 10 (15.2%) | 1.024 (0.670-1.567) | 1.137 (0.738-1.751) |
| DOL 6 | 55 (83.3%) | 11 (16.7%) | 1.226 (0.764-1.967) | 1.159 (0.702-1.913) |
| DOL 7 | 57 (86.4%) | 9 (13.6%) | 1.270 (0.747-2.161) | 1.175 (0.682-2.026) |
| DOL 8 | 48 (72.7%) | 18 (27.3%) | 0.815 (0.558-1.192) | 0.816 (0.576-1.156) |
| DOL 9 | 48 (72.7%) | 18 (27.3%) | 1.244 (0.883-1.752) | 1.146 (0.849-1.546) |
| DOL 10 | 47 (71.2%) | 19 (28.8%) | 0.795 (0.513-1.231) | 0.835 (0.587-1.189) |
| DOL 11 | 38 (57.6%) | 28 (42.4%) | 1.066 (0.683-1.662) | 1.081 (0.718-1.630) |
| DOL 12 | 35 (53%) | 31 (47%) | 1.066 (0.723-1.572) | 1.012 (0.742-1.380) |
| DOL 13 | 33 (50%) | 33 (50%) | 1.167 (0.779-1.748) | 1.074 (0.780-1.479) |
| DOL 14 | 33 (50%) | 33 (50%) | 0.955 (0.623-1.466) | 0.888 (0.628-1.256) |

Subgroup analysis examining the association between metabolic acidosis and adverse outcomes in extremely low gestational age neonates (ELGANs; <28 weeks’ gestation). No statistically significant associations were observed in either model across the 14-day postnatal period.
*IPTW, Inverse Probability of Treatment Weighting; **DOL, Day(s) of life

**Supplementary Table 5. Subgroup Analysis: Association Between Metabolic Acidosis and Outcome in 28-31 Weeks' GA)**

| Acidosis Occurence | **With Acidosis**  **n (%)** | **Without Acidosis**  **n (%)** | **Unweighted Model(GLM)**  **OR (95% CI)** | | **IPTW* Model**  **OR (95% CI)** |
| --- | --- | --- | --- | --- | --- |
| DOL** 1 | 40 (39.2%) | 62 (60.8%) | 0.936 (0.802-1.093) | 0.936 (0.822-1.066) | |
| DOL 2 | 42 (41.2%) | 60 (58.8%) | 1.029 (0.884-1.197) | 0.922 (0.814-1.045) | |
| DOL 3 | 76 (74.5%) | 26 (25.5%) | 0.889 (0.740-1.068) | 0.870 (0.688-1.101) | |
| DOL 4 | 82 (80.4%) | 20 (19.6%) | 0.921 (0.746-1.136) | 0.991 (0.861-1.141) | |
| DOL 5 | 78 (76.5%) | 24 (23.5%) | 0.993 (0.793-1.244) | 1.048 (0.905-1.213) | |
| DOL 6 | 65 (63.7%) | 37 (36.3%) | 1.123 (0.895-1.410) | **1.226 (1.015-1.482)** | |
| DOL 7 | 45 (44.1%) | 57 (55.9%) | 0.885 (0.726-1.079) | 0.844 (0.710-1.004) | |
| DOL 8 | 38 (37.3%) | 64 (62.7%) | 0.875 (0.678-1.129) | 0.839 (0.724-0.973) | |
| DOL 9 | 27 (26.5%) | 75 (73.5%) | 1.291 (0.919-1.815) | **1.263 (1.024-1.559)** | |
| DOL 10 | 21 (20.6%) | 81 (79.4%) | 1.183 (0.863-1.621) | 1.270 (0.978-1.650) | |
| DOL 11 | 22 (21.6%) | 80 (78.4%) | 0.871 (0.634-1.196) | 0.785 (0.593-1.038) | |
| DOL 12 | 19 (18.6%) | 83 (81.4%) | 1.124 (0.790-1.599) | 1.096 (0.903-1.329) | |
| DOL 13 | 16 (15.7%) | 86 (84.3%) | 0.949 (0.669-1.346) | 0.957 (0.817-1.121) | |
| DOL 14 | 13 (12.7%) | 89 (87.3%) | 1.028 (0.730-1.446) | 0.998 (0.793-1.256) | |

Subgroup analysis assessing the association between metabolic acidosis and adverse outcomes in preterm infants born at 28–31 weeks’ gestation. Significant associations were observed in the IPTW model on DOL 6, DOL 8, and DOL 9, suggesting that postnatal metabolic acidosis during specific time points may have clinical relevance in this gestational age group.
*IPTW, Inverse Probability of Treatment Weighting; **DOL, Day(s) of life

**Supplementary Table 6. Association Between Metabolic Acidosis and Daily Fluid Intake**

|  | **Metabolic Acidosis** | | | **Severe Metabolic Acidosis** | | |
| --- | --- | --- | --- | --- | --- | --- |
|  | **Yes** | **No** | ***p*-value** | **Yes** | **No** | ***p*-value** |
| Daily Fluid Intake (mL/kg/day), median (IQR) | 150  (129-166) | 145  (129-157) | <0.001 | 153  (135-171) | 147  (129-161) | 0.005 |

Daily fluid intake is presented as median and interquartile range (IQR). Metabolic acidosis was defined as pH < 7.35 with either base excess < –4 mmol/L or bicarbonate < 18 mmol/L and severe metabolic acidosis was defined as pH < 7.20 with either base excess < –10 mmol/L or bicarbonate < 12 mmol/L. Both outcomes were analyzed as binary variables (presence vs. absence). Group differences in fluid intake were assessed using the Wilcoxon rank-sum test.

**Supplementary Figure 1. Association Between Metabolic Acidosis and Daily Fluid Intake**


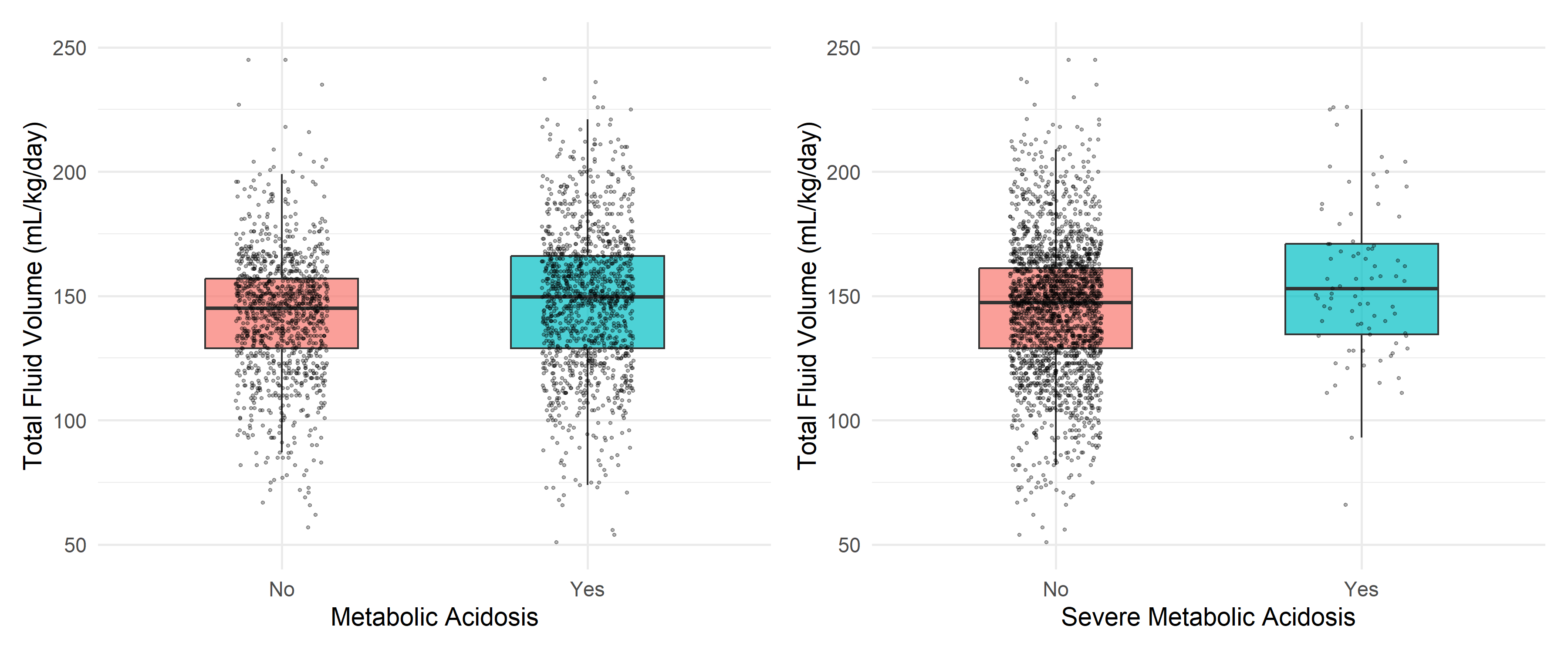


Boxplots comparing total fluid volume (mL/kg/day) between days with and without metabolic acidosis (left) and severe metabolic acidosis (right). Daily fluid intake was significantly higher on days with metabolic acidosis (median [IQR]: 150 [129–166] vs. 145 [129–157], *p* < 0.001) and severe metabolic acidosis (153 [135–171] vs. 147 [129–161], *p* = 0.005). Metabolic acidosis was defined as pH < 7.35 with either base excess < –4 mmol/L or bicarbonate < 18 mmol/L, and severe metabolic acidosis was defined as pH < 7.20 with either base excess < –10 mmol/L or bicarbonate < 12 mmol/L.
